# Diagnostic Performance of Magnifying Endoscopy for *Helicobacter pylori* Infection: A Meta-Analysis

**DOI:** 10.1371/journal.pone.0168201

**Published:** 2016-12-19

**Authors:** Qingqing Qi, Chuanguo Guo, Rui Ji, Zhen Li, Xiuli Zuo, Yanqing Li

**Affiliations:** Department of Gastroenterology, Qilu Hospital, Shandong University, Jinan, Shandong Province, China; University of Utah Health Sciences Center, UNITED STATES

## Abstract

**Background:**

Diagnosis of *Helicobacter pylori* (*H*. *pylori*) infection using magnifying endoscopy offers advantages over conventional invasive and noninvasive tests.

**Objective:**

This meta-analysis aimed to assess the diagnostic performance of magnifying endoscopy in the prediction of *H*. *pylori* infection.

**Methods:**

A literature search of the PubMed, Medline, EMBASE, Science Direct and the Cochrane Library databases was performed. A random-effects model was used to calculate the diagnostic efficiency of magnifying endoscopy for *H*. *pylori* infection. A summary receiver operator characteristic curve was plotted, and the area under the curve (AUC) was calculated.

**Results:**

A total of 18 studies involving 1897 patients were included. The pooled sensitivity and specificity of magnifying endoscopy to predict *H*. *pylori* infection were 0.89 [95% confidence interval (CI) 0.87–0.91] and 0.82 (95%CI 0.79–0.85), respectively, with an AUC of 0.9461. When targeting the gastric antrum, the pooled sensitivity and specificity were 0.82 (95%CI 0.78–0.86) and 0.72 (95%CI 0.66–0.78), respectively. When targeting the gastric corpus, the pooled sensitivity and specificity were 0.92 (95%CI 0.90–0.94) and 0.86 (95%CI 0.82–0.88), respectively. The pooled sensitivity and specificity using magnifying white light endoscopy were 0.90 (95%CI 0.87–0.91) and 0.81 (95%CI 0.77–0.84), respectively. The pooled sensitivity and specificity using magnifying chromoendoscopy were 0.87 (95%CI 0.83–0.91) and 0.85 (95%CI 0.80–0.88), respectively. The “pit plus vascular pattern” classification in the gastric corpus observed by magnifying endoscopy was able to accurately predict the status of *H*. *pylori* infection, as indicated by a pooled sensitivity and specificity of 0.96 (95%CI 0.94–0.97) and 0.91 (95%CI 0.87–0.93), respectively, with an AUC of 0.9872.

**Conclusions:**

Magnifying endoscopy was able to accurately predict the status of *H*. *pylori* infection, either in magnifying white light endoscopy or magnifying chromoendoscopy mode. The “pit plus vascular pattern” classification in the gastric corpus is an optimum diagnostic criterion.

## Introduction

*Helicobacter pylori* (*H*. *pylori*) infection is a well-described risk factor for gastritis, peptic ulcer, gastric mucosa-associated lymphoid tissue lymphoma and gastric adenocarcinoma [1–3]. Several diagnostic tests for the presence of this bacterium have been widely used in clinical practice. However, each of these tests has certain disadvantages [4]. Noninvasive tests (e.g., serology, urea breath test, or stool test) are convenient and accurate. However, these tests do not provide real-time information on the gastric mucosa, which is clinically important, especially for patients with special indications, such as symptoms of dyspepsia, a family history of cancer, etc. For these patients, an endoscopic examination is necessary to directly describe the gastric diseases and identify more precancerous lesions in a timely manner. However, the invasive tests (e.g., histology, culture, or rapid urease test) used for *H*. *pylori* examination during the endoscopic examination require biopsy samples, which will lead to unnecessary injury and medical costs. Moreover, random biopsies are not always sufficiently accurate for the detection of *H*. *pylori* due to its focal distribution.

Previous studies conclude that it is not feasible to diagnose *H*. *pylori*-related gastritis based on conventional endoscopic findings. Features labeled as gastritis poorly correlate with histopathological results, and interobserver agreement is poor for the endoscopic features of gastritis [5–7]. Magnifying endoscopy allows for the clear visualization of the microsurface structure and microvascular architecture of the gastric mucosa. Recently, different features observed using magnifying endoscopy have been demonstrated to describe the presence of *H*. *pylori*. Prospective studies have been carried out to explore the usefulness of magnifying endoscopy for the diagnosis of *H*. *pylori* infection. Almost all of these studies selected the gastric antrum or corpus as the observed site. However, the optimum site to observe for the endoscopic diagnosis of *H*. *pylori* infection has not yet been identified. Moreover, the optimum diagnostic classification is also needed to confirm among various endoscopic criteria.

Thus, the aim of our study was to perform a meta-analysis of published data to assess the diagnostic performance of magnifying endoscopy for *H*. *pylori* infection.

## Materials and Methods

### Search strategy

We systematically searched the PubMed, Medline, EMBASE, Science Direct and the Cochrane Library databases to identify all relevant articles published until August 2015. The following search terms were used: “*Helicobacter pylori*”, “*H*. *pylori*”, “HP”, “gastritis” AND “magnifying”, “magnification”, “magnified”, “zoom”. The references in the available articles and reviews were also carefully examined to avoid missing studies. After scanning the titles and abstracts of articles selected from the initial search, we read the full texts of eligible articles. Two investigators independently searched the articles, and disagreements were resolved by discussion. Our meta-analysis was performed according to the PRISMA statement [8].

### Selection of studies

The inclusion criteria were as follows:

1. Magnifying endoscopy was used for the diagnosis of *H*. *pylori* infection;
2. The numbers of true-positive (TP), false-positive (FP), true-negative (TN) and false-negative (FN) cases were reported or could be calculated from the study to construct 2×2 tables;
3. At least one of rapid urease test, urea breath test, *H*. *pylori* culture or histopathological examination was applied as the reference standard;
4. Studies that were published as full articles in English language.

The exclusion criteria were as follows:

1. Studies without a definite reference standard;
2. Studies without complete data for constructing 2×2 tables with TP, FP, FN and TN;
3. Studies that overlapped the studies selected;
4. Studies that included patients with a history of *H*. *pylori* eradication therapy or using proton pump inhibitors;
5. Review articles, case reports, editorials, expert opinions, comments, letters to the editor, and meeting abstracts.

### Assessment of study quality

The Quality Assessment of Diagnostic Accuracy Studies-2 (QUADAS-2) tool was used to assess the quality and risk of bias of all included studies [9]. This tool consists of four key domains: patient selection, index test, reference standard and flow and timing. Each domain is assessed in terms of the risk of bias, and the first three are also assessed in terms of concerns regarding applicability. Both the risk of bias and the concerns regarding applicability are rated as ‘‘low”, ‘‘high” or ‘‘unclear”. Signaling questions were answered to help us make a judgment. If the study was judged as “low” on all domains, it would be judged as a “low risk of bias” or “low concern regarding applicability”. In contrast, it would be judged as having a “risk of bias” or having “concerns regarding applicability”, if the study was judged as “high” in one or more domains. The “unclear” was used when a judgment was difficult to make due to insufficient data. The assessment procedure was performed and crosschecked by two independent reviewers.

### Data extraction

The following information was obtained from each study: the first author, year of publication, country, number of patients, age and sex ratio, endoscopy type, endoscopy mode, magnification factor, observed site and diagnostic classification. The numbers of TP, FP, TN and FN were also extracted, and 2×2 tables were constructed. All data were extracted independently by two investigators, and discrepancies were resolved by discussion.

### Statistical analysis

Cochran’s Q and inconsistency (I^2^) were measured to estimate the heterogeneity of included studies. The Spearman correlation coefficient was calculated to test heterogeneity due to threshold effect. A random-effects model was used for the meta-analysis because the group of studies in our research is a random sample of all possible studies. The pooled sensitivity, specificity, positive likelihood ratio (LR+), negative likelihood ratio (LR-) and diagnostic odds ratio (DOR) as well as the corresponding 95% confidence interval (CI) were calculated. A summary receiver operator characteristic (SROC) curve was plotted, and the area under the curve (AUC) was calculated. We performed subgroup analyses to assess the diagnostic performance of magnifying endoscopy according to different features in selected studies. Deeks’ funnel plot was used to assess the potential publication bias of included studies. Meta-DiSc (version 1.4) and Stata (version 12.1) were used to perform the meta-analysis. *P*<0.05 was considered statistically significant.

## Results

### Selection of studies

**Fig 1:**
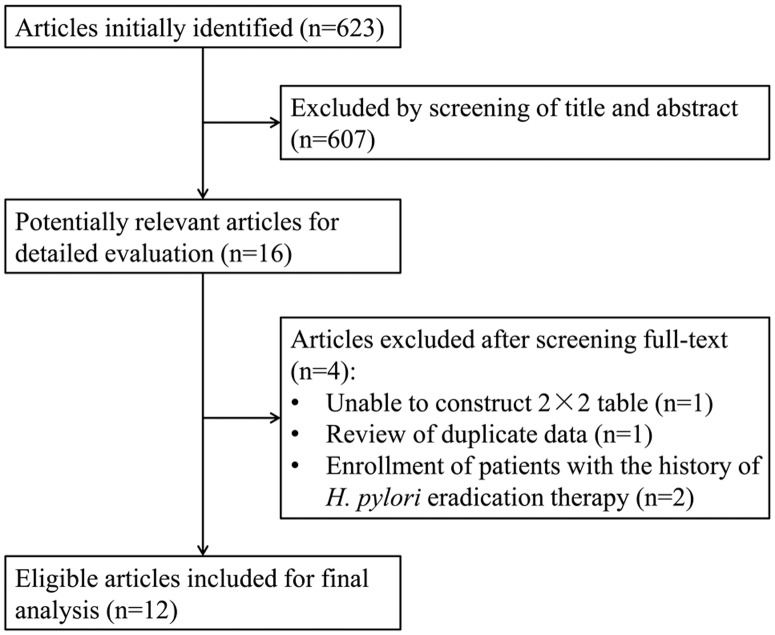
Flow diagram of the study selection process for the meta-analysis. Finally, 18 studies were identified from the 12 articles.

After the primary search, a total of 623 studies were identified. Fig 1 shows the selection process and reasons for exclusion. After reviewing the full text, articles were excluded due to the following reasons: unable to construct a 2×2 table (n = 1) [10], review of duplicate data (n = 1) [11] and the enrollment of patients with the history of *H*. *pylori* eradication treatment (n = 2) [12, 13]. Finally, 12 articles [14–25] were selected for the meta-analysis. After a careful review of the full articles, three, four and two separate studies were identified from the articles by Nakagawa S [16], Gonen C [21] and Qi QQ [23], respectively. These studies identified from one article were performed with different magnifying endoscopy modes, or observed different gastric sites. In total, 18 eligible studies were identified from the 12 included articles. All of these studies met the inclusion criteria. All included studies were prospective trials.

The main characteristics of the included studies are summarized in Table 1. In total, 1897 patients were enrolled, with a mean number of 105 patients per study. Data to evaluate the diagnostic performance of magnifying endoscopy for *H*. *pylori* infection were extracted from these studies.

**Table 1:** Characteristics of the included studies.

Study	Country	Number of patients	Mean age (years)	M/F	Endoscopy type	Endoscopy mode	Magnification factor	Observed site	Diagnostic classification
Yagi K et al. 2002 [14]	Japan	94	—	—	Olympus, GIF-Q240Z	WLE	×80	Antrum	wDRP, iDRP
Yagi K et al. 2002 [15]	Japan	297	47	145/152	Olympus, GIF-Q240Z	WLE	×80	Corpus	Z0-3
Nakagawa S et al. 2003 [16]	Japan	92	55.6	56/36	Olympus, GIF-Q240Z	WLE	×80	Antrum/Corpus	R.I.O
Yang JM et al. 2003 [17]	China	140	50.6	68/72	Olympus, GIF-Q240Z	WLE	×80	Corpus	R.I.D
Egi Y et al. 2006 [18]	Japan	44	—	—	Fujinon, EG-450ZW5	indigo carmine	×100	Cardia	Fundic and nonfundic type
Anagnostopoulos GK et al. 2007 [19]	UK	95	58.6	52/43	Olympus, GIF-Q240Z	WLE	×115	Corpus	Type1-3
Bansal A et al. 2008 [20]	USA	47	65	46/1	Olympus, GIF 240Z	NBI	×115	Antrum and corpus	irregular pattern with decreased density of vessels
Gonen C et al. 2009 [21]	Turkey	129	48.6	32/97	Fujinon, EG-490ZW5	WLE/indigo carmine	×100	Corpus/Antrum	Z0-3/CZ0-2/wDRP, iDRP/CZA0-2
Tahara T et al. 2009 [22]	Japan	106	58.7	64/42	Olympus, GIF-H260Z	NBI	×85	Corpus	Normal+Type1-3
Qi QQ et al. 2013 [23]	China	84	49.3	47/37	Pentax, EG-2990Zi	WLE/i-scan	×100	Corpus	Type1-3
Kawamura M et al. 2013 [24]	Japan	175	63.9	116/59	Olympus, Model Q240Z or H260Z	WLE	—	Corpus	COs
Liu H et al. 2014 [25]	China	90	57.5	49/41	Olympus, Model GIF H260Z	NBI	×70	Antrum	Type A-E

All included studies are prospective trials. WLE, white light endoscopy; NBI, narrow band imaging. The details of different diagnostic classifications is listed in S1 Text.

The studies were carried out in Japan [14–16, 18, 22, 24], China [17, 23, 25], Turkey [21], the USA [20] and the UK [19]. In 5 studies [14, 16, 21, 25], the endoscopic features of the gastric autumn were investigated to diagnose *H*. *pylori* infection. Another 11 studies [15–17, 19, 21–24] selected the gastric body as the observed site, and most of these studies used the Z0-3 [15, 21], Type1-3 [19, 23] or normal along with Type1-3 [22] classifications as the endoscopic diagnostic criteria (n = 6). We herein describe these three classifications as a “pit plus vascular pattern” because all of them combined the appearance of gastric pits, collecting venules and subepithelial capillary network (SECN) into their classifications. The detailed information of all endoscopic classifications is listed in S1 Text. Moreover, 11 studies [14–17, 19, 21, 23, 24] used magnifying white light endoscopy (WLE) and another 7 studies used magnifying chromoendoscopy (indigo carmine [18, 21], NBI [20, 22, 25] and i-scan [23]). Most studies used Olympus endoscopes [14–17, 19, 20, 22, 24, 25], and the remaining studies used Fujinon [18, 21] or Pentax [23] endoscopes.

### Quality assessment

**Fig 2:**
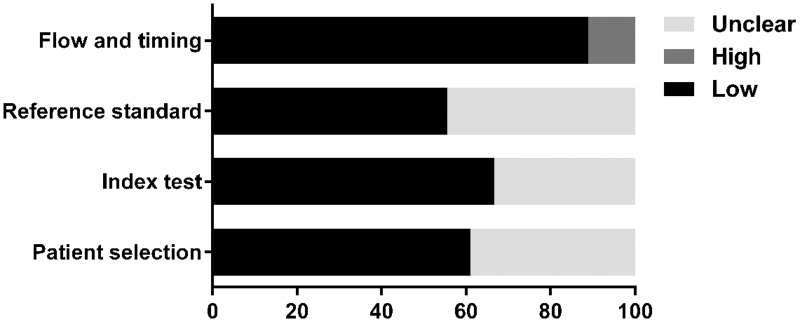
Proportion of studies with low, high, or unclear risk of bias, %.

**Fig 3:**
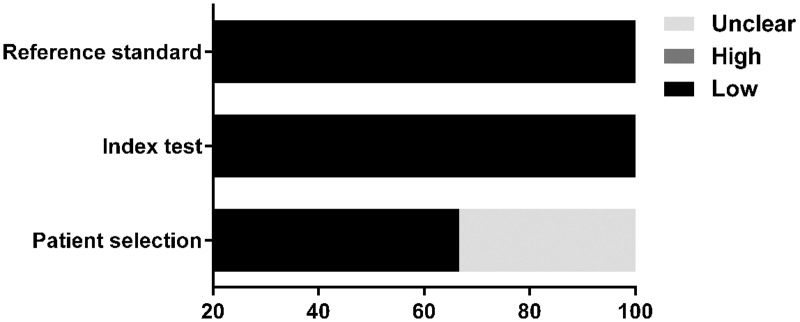
Proportion of studies with low, high, or unclear concerns regarding applicability, %.

The quality of eligible studies according to the QUADAS-2 criteria was evaluated as shown in Table 2 and is graphically displayed in Figs 2 and 3. Generally, the included studies met most of the quality criteria. However, some studies did not clearly indicate whether consecutive or random patients were enrolled. Moreover, blind assessment between endoscopic diagnoses and reference standards was not explicitly stated in some studies. These factors may introduce bias.

**Table 2:** The Quality Assessment of Diagnostic Accuracy Studies-2 (QUADAS-2) tool for quality assessment of studies selected for the meta-analysis.

Study	Risk of bias				Applicability concerns		
	Patient selection	Index test	Reference standard	Flow and timing	Patient selection	Index test	Reference standard
Yagi K et al. 2002 [14]	?	☺	?	☺	?	☺	☺
Yagi K et al. 2002 [15]	?	?	☺	☺	?	☺	☺
Nakagawa S et al. 2003 [16] a	☺	☺	☺	☺	☺	☺	☺
Nakagawa S et al. 2003 [16] a	☺	☺	☺	☺	☺	☺	☺
Nakagawa S et al. 2003 [16] a	☺	☺	☺	☺	☺	☺	☺
Yang JM et al. 2003 [17]	?	?	?	☺	?	☺	☺
Egi Y et al. 2006 [18]	☺	☺	?	☹	☺	☺	☺
Anagnostopoulos GK et al. 2007 [19]	☺	?	☺	☺	?	☺	☺
Bansal A et al. 2008 [20]	?	?	☺	☺	?	☺	☺
Gonen C et al. 2009 [21] b	?	☺	?	☺	☺	☺	☺
Gonen C et al. 2009 [21] b	?	☺	?	☹	☺	☺	☺
Gonen C et al. 2009 [21] b	☺	☺	?	☺	☺	☺	☺
Gonen C et al. 2009 [21] b	☺	☺	?	☺	☺	☺	☺
Tahara T et al. 2009 [22]	?	?	☺	☺	☺	☺	☺
Qi QQ et al. 2013 [23] c	☺	☺	☺	☺	☺	☺	☺
Qi QQ et al. 2013 [23] c	☺	☺	☺	☺	☺	☺	☺
Kawamura M et al. 2013 [24]	☺	?	?	☺	☺	☺	☺
Liu H et al. 2014 [25]	☺	☺	☺	☺	?	☺	☺

☺Low Risk; ☹High Risk;? Unclear Risk. a, b, c indicate studies identified from one article.

### Diagnostic performance of magnifying endoscopy for *H*. *pylori* infection

**Fig 4:**
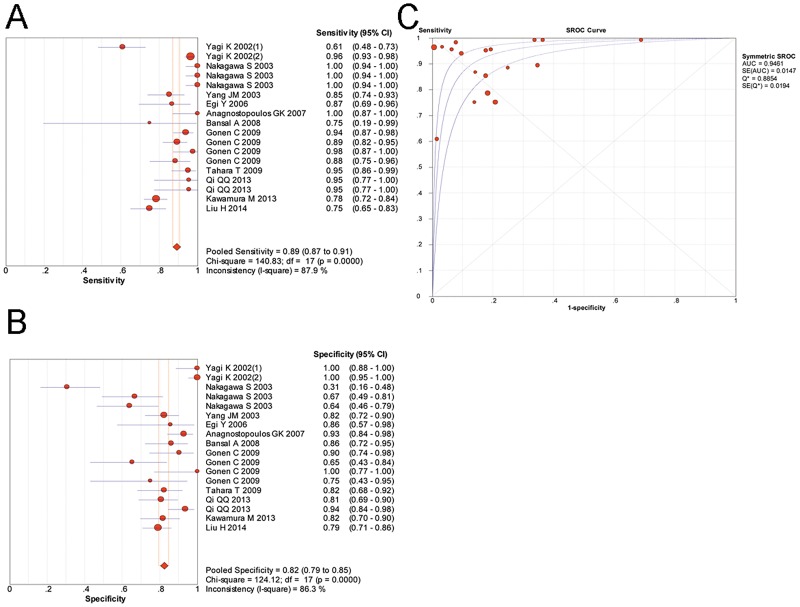
The diagnostic performance of magnifying endoscopy in predicting *H*. *pylori* infection. (A), pooled sensitivity; (B), pooled specificity; (C), summary receiver operating characteristic curve for diagnosis by magnifying endoscopy. CI, confidence interval; AUC, area under the curve; SE, standard error.

A random-effects model was used in our meta-analysis of the included 18 studies and the results are shown in Fig 4 and Table 3. The pooled sensitivity of magnifying endoscopy to predict *H*. *pylori* infection was 0.89 (95%CI 0.87–0.91), and the pooled specificity was 0.82 (95%CI 0.79–0.85). The LR+, LR- and DOR were 5.16 (95%CI 3.32–8.02), 0.11 (95%CI 0.07–0.18) and 59.58 (95%CI 30.63–115.88), respectively. The area under the SROC curve was 0.9461 (SE = 0.0147), indicating a high level of diagnostic accuracy of magnifying endoscopy for *H*. *pylori* infection.

**Table 3:** Diagnostic performance of magnifying endoscopy for *H*. *pylori* infection.

Study group	Number of studies	Diagnostic performance						I^2^ for DOR
		Sensitivity 95%CI	Specificity 95%CI	LR+ 95%CI	LR- 95%CI	DOR 95%CI	AUC	
All	18	0.89 (0.87–0.91)	0.82 (0.79–0.85)	5.16 (3.32–8.02)	0.11 (0.07–0.18)	59.58 (30.63–115.88)	0.9461	67.10
Observed site								
Antrum	5	0.82 (0.78–0.86)	0.72 (0.66–0.78)	2.99 (1.39–6.44)	0.25 (0.15–0.40)	14.72 (8.86–24.47)	0.8611	0.00
Corpus	11	0.92 (0.90–0.94)	0.86 (0.82–0.88)	5.96 (3.88–9.15)	0.06 (0.03–0.13)	127.38 (48.29–336.05)	0.9702	70.60
Diagnostic criterion								
“pit plus vascular pattern” in corpus	6	0.96 (0.94–0.97)	0.91 (0.87–0.93)	9.56 (4.72–19.34)	0.05 (0.03–0.08)	203.69 (77.11–538.02)	0.9872	29.70
Endoscopy mode								
White light endoscopy	11	0.90 (0.87–0.91)	0.81 (0.77–0.84)	4.88 (2.63–9.08)	0.09 (0.04–0.19)	75.28 (30.11–188.21)	0.9576	68.60
Chromoendoscopy	7	0.87 (0.83–0.91)	0.85 (0.80–0.88)	5.44 (3.54–8.35)	0.13 (0.06–0.26)	46.52 (15.68–138.03)	0.9238	67.20

LR+, positive likelihood ratio; LR-, negative likelihood ratio; DOR, diagnostic odds ratio; AUC, area under the curve; CI, confidence interval.

Subgroup analyses were performed according to different study features, and all related results are presented in Table 3. In 5 studies, the gastric antrum was observed to predict the status of *H*. *pylori* infection. The pooled sensitivity and specificity were 0.82 (95%CI 0.78–0.86) and 0.72 (95%CI 0.66–0.78), respectively. The DOR was 14.72 (95%CI 8.86–24.47). The AUC was 0.8611. Another 11 studies evaluated the features of the gastric corpus as indicators of *H*. *pylori* infection. The pooled sensitivity and specificity were 0.92 (95%CI 0.90–0.94) and 0.86 (95%CI 0.82–0.88), respectively. The DOR was 127.38 (95%CI 48.29–336.05). The AUC was 0.9702, suggesting a much higher diagnostic accuracy compared with studies of the gastric antrum.

**Fig 5:**
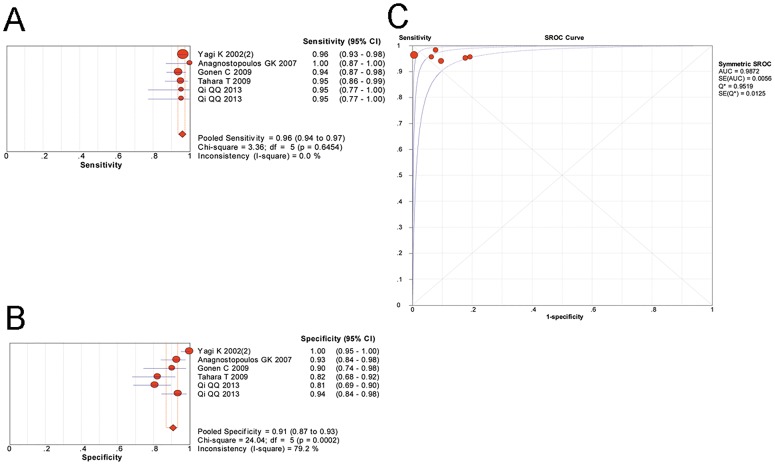
The diagnostic performance of a “pit plus vascular pattern” classification in the gastric corpus by magnifying endoscopy in predicting *H*. *pylori* infection. (A), pooled sensitivity; (B), pooled specificity; (C), summary receiver operating characteristic curve for diagnosis by magnifying endoscopy. CI, confidence interval; AUC, area under the curve; SE, standard error.

Furthermore, in studies selecting the gastric corpus as the observed site, the most commonly used diagnostic criteria were the “pit plus vascular pattern” classification as described above. A meta-analysis of these 6 studies showed that the pooled sensitivity and specificity were 0.96 (95%CI 0.94–0.97) and 0.91 (95%CI 0.87–0.93), respectively. The LR+, LR- and DOR were 9.56 (95%CI 4.72–19.34), 0.05 (95%CI 0.03–0.08) and 203.69 (95%CI 77.11–538.02), respectively. The AUC was 0.9872 (SE = 0.0056), indicating that typical features of gastric corpus observed by magnifying endoscopy were able to accurately predict the status of *H*. *pylori* infection. (Fig 5)

We also performed an analysis according to the different endoscopic modes used in the studies. The pooled sensitivity and specificity obtained from the analysis of studies using magnifying WLE mode were 0.90 (95%CI 0.87–0.91) and 0.81 (95%CI 0.77–0.84), respectively. The AUC was 0.9576. Similar diagnostic performance of magnifying chromoendoscopy (indigo carmine, NBI and i-scan) was found compared with magnifying WLE, with a sensitivity, specificity and AUC of 0.87 (95%CI 0.83–0.91), 0.85 (95%CI 0.80–0.88) and 0.9238, respectively.

### Heterogeneity test

A high heterogeneity was observed among all studies, with an I^2^ value of 67.10% for the DOR. The Spearman correlation coefficient was 0.225 (*P* = 0.370), suggesting no heterogeneity caused by threshold effect. Subgroup analyses were performed to identify the potential source of heterogeneity and we principally assessed the effect of observed sites and endoscopic modes. All subgroup analyses showed high heterogeneity, with an I^2^>50% for the DOR, except for the antrum subgroup (I^2^ = 0.00). In addition, an analysis of studies using the “pit plus vascular pattern” classification in the corpus showed reduced heterogeneity compared with overall studies, with the I^2^ for the DOR decreased from 67.10% to 29.70%. (Table 3)

### Publication bias estimate

**Fig 6:**
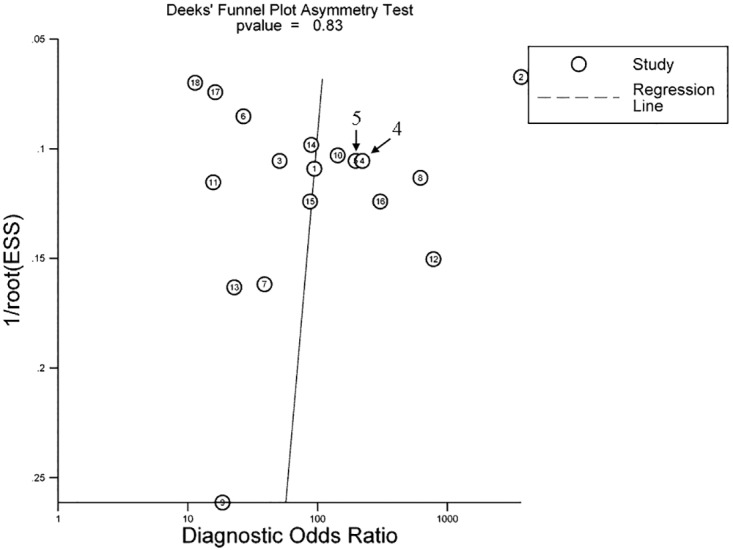
Deeks’ funnel plot to evaluate publication bias. *P* = 0.83 indicates a symmetrical funnel shape and suggests that publication bias is absent.

Deek’ funnel plot was used to assess the potential publication bias of the studies in our meta-analysis. Fig 6 shows a symmetrical funnel shape (*P* = 0.83), indicating that there was no striking publication bias in this study.

## Discussion

Our present meta-analysis included all available studies to evaluate the diagnostic performance of magnifying endoscopy in predicting *H*. *pylori* infection. We confirmed that endoscopy with magnification was able to accurately indicate the status of *H*. *pylori* infection, in both magnifying WLE or chromoendoscopy mode. Furthermore, the diagnostic efficiency was better when targeting the gastric corpus than the antrum, and a “pit plus vascular pattern” classification in the corpus was the optimum diagnostic criteria. Our results provide significant information to both clinicians and patients. Magnifying endoscopy is an accurate alternative method for the diagnosis of *H*. *pylori* infection, with a diagnostic efficiency similar to that of traditional methods (e.g., invasive and noninvasive tests).

*H*. *pylori* infection is commonly accepted as a predisposing factor for the development of gastric diseases [2, 26]. Therefore, its timely and accurate diagnosis is necessary in clinical practice. In addition to traditional invasive and noninvasive tests, endoscopy has also been used as a diagnostic tool for *H*. *pylori* infection for many years. However, the features observed by conventional endoscopy are insufficiently accurate for the diagnosis of *H*. *pylori* gastritis [6, 27]. Magnifying endoscopy has been developed to clearly visualize the superficial gastric mucosal and capillary patterns. A number of studies that describe the special features of normal *H*. *pylori*-negative and abnormal *H*. *pylori*-positive gastric mucosa using this type of endoscopy have been published. Our meta-analysis of these studies provides strong evidence for the endoscopic diagnosis of *H*. *pylori* infection with magnification function, with an AUC of 0.9461 for all included studies.

To obtain more detailed information on the gastric mucosa, chromoendoscopy was applied using either topically sprayed dyes (e.g., indigo carmine, methylene blue, or acetic acid), or electronic staining (e.g., NBI and i-scan). Both chromoendoscopy methods are useful in the detection and diagnosis of gastric lesions. However, limitations exist as spraying dyes is time-consuming [28, 29], and performance of electronic staining need more experience for endoscopists [30]. In our analysis, 11 studies used magnifying WLE [14–17, 19, 21, 23, 24] and the remaining 7 studies used magnifying chromoendoscopy [18, 20–23, 25]. In our previous study, the diagnostic accuracy of i-scan was better than that of WLE [23]. However, meta-analysis of studies that separately used these different modes yielded a pooled sensitivity, specificity and AUC of 0.90 (95%CI 0.87–0.91), 0.81 (95%CI 0.77–0.84) and 0.9576, respectively, for magnifying WLE, and 0.87 (95%CI 0.83–0.91), 0.85 (95%CI 0.80–0.88), and 0.9238, respectively, for magnifying chromoendoscopy. These results suggest that the diagnostic potential of the two magnifying endoscopy modes is similar for the diagnosis of *H*. *pylori* infection. This finding may be explained by the fact that WLE mode is generally integrated with high resolution endoscopy, which could describes the gastric pit and vascular pattern in sufficient detail for the diagnosis of *H*. *pylori* infection. Therefore, the WLE mode seems to be the better choice for the endoscopic diagnosis of *H*. *pylori* infection because it features a shorter examination time, lower cost and requires less experience. However, this conclusion needs to be supported by further well-designed clinical trials due to the high heterogeneity among the included studies.

The magnifying endoscopic appearances are a reflection of the histological features of the structure of the gastric mucosa. The normal mucosa in the corpus is comprised of regular small round pits, which are surrounded by a honeycomb-type network of SECN intermingled with regularly arranged collecting venules [20]. In contrast, the antral glands are more tortuous and branched than glands in the corpus, with coil-shaped SECN [31, 32]. In *H*. *pylori-*induced gastritis, edema as well as the destruction and regeneration of vessels caused by inflammation enlarges the pits and makes the capillaries and venules irregular or invisible. However, collecting venules are located in deeper layers in the gastric antrum and cannot be visualized by magnifying endoscopy [19, 20]. Therefore, the appearance of collecting venules cannot be used as a typical feature to judge the status of *H*. *pylori* in the antrum. This characteristic might explain the higher diagnostic accuracy of endoscopic criteria in the gastric corpus compared with the antrum in our present meta-analysis. Because the pooled sensitivity [0.92 (95%CI 0.90–0.94) vs. 0.82 (95%CI 0.78–0.86)], specificity [0.86 (95%CI 0.82–0.88) vs. 0.72 (95%CI 0.66–0.78)] and AUC (0.9702 vs. 0.8611) were higher in the gastric corpus than in the antrum, we conclude that the gastric corpus is a better observed site for the diagnosis of *H*. *pylori* using magnifying endoscopy. Moreover, the endoscopic appearance of the gastric mucosa in response to alcohol, nonsteroidal anti-inflammatory drugs and autoimmune dysfunction is similar to that due to *H*. *pylori* infection, which hinders the distinguishing of these conditions only based on magnifying endoscopy. However, these conditions caused by other etiological factors are relatively uncommon compared with *H*. *pylori* infection, and a detailed medical history could help to distinguish them from *H*. *pylori* infection.

Moreover, several different endoscopic criteria were used in these clinical trials, even in the gastric corpus. The most popular criteria are Z0-3 [15, 21], Type1-3 [19, 23] or normal along with Type1-3 [22] classifications. All these criteria classified the mucosa of the gastric corpus into 3 or 4 types based on the appearance of gastric pits, SECN and collecting venules. We collectively describe all these 3 classifications as a “pit plus vascular pattern” in this present study. In summary, the typical pattern used to predict an *H*. *pylori* negative gastric mucosa is featured as honeycomb−type SECN with regularly arrangement of collecting venules and regular, round pits. In the *H*. *pylori* positive gastric mucosa, the collecting venules become invisible and gastric pits are enlarged, with SECN irregular or disappeared. Based on this classification, the pooled sensitivity, specificity and AUC were as high as 0.96 (95%CI 0.94–0.97), 0.91 (95%CI 0.87–0.93) and 0.9872, respectively. These values almost match the sensitivity and specificity of any diagnostic tests in use for the diagnosis of *H*. *pylori* including urea breath test, rapid urease test, serology and histologic examination [33]. Furthermore, compared with overall studies, the I^2^ for DOR of studies using a “pit plus vascular pattern” classification significantly decreased from 67.10% to 29.70%, indicating a much lower heterogeneity. All these results suggest that the infection of *H*. *pylori* could be accurately predicted using a “pit plus vascular pattern” classification in the gastric corpus observed by magnifying endoscopy.

There are several limitations in our study. First, some studies were identified from the same article. These studies examined the same population but employed different modes or observed different sites. We included all of these studies in our analysis, which might influence the independence of included studies. However, excluding any one of these studies is inappropriate due to possible selection bias. In addition, we repeated all related analyses and found no obvious changes in the pooled diagnostic results after removing any one or all of these studies. Second, all studies used conventional diagnostic tests (rapid urease test, urea breath test, *H*. *pylori* culture or histopathological examination, etc.) as the reference standard for *H*. *pylori* infection. However, in some studies, *H*. *pylori* infection was confirmed only if two or more test results were positive, whereas in other studies just one test was referred. Although this factor might introduce bias, we could believe that prediction of *H*. *pylori* infection using magnifying endoscopy is at least as accurate as one of the currently invasive and noninvasive tests. Moreover, urea breath test will be positive for subjects with antral gastritis and without *H*. *pylori* in the corpus, while it is difficult for the detection of *H*. *pylori* in the corpus when target gastric corpus using magnifying endoscopy. However, in this case the gastric antrum should be observed because subjects with antral gastritis have featured appearance in antrum under magnifying endoscopy, which could help to predict the status of *H*. *pylori* infection. Third, heterogeneity existed across the 18 included studies. Although the I^2^ for DOR significantly decreased to 0% and 29.7% in the subgroup analysis of the gastric antrum and “pit plus vascular pattern” classification, heterogeneity persisted in other subgroup analyses. More high-quality clinical trials are needed to confirm our findings. Finally, we only calculated the pooled diagnostic efficiency of the “pit plus vascular pattern” classification in the subgroup analysis. Other classifications were not analyzed due to a lack of sufficient related studies. Further studies using these classifications should be carried out to address this problem.

In conclusion, magnifying endoscopy displays high diagnostic potential in the prediction of *H*. *pylori* infection. The “pit plus vascular pattern” classification in the gastric corpus is an optimum endoscopic criterion for *H*. *pylori* infection in clinical practice.

## Supporting Information

S1 Checklist PRISMA Checklist. (DOC)

S1 Text The details of different diagnostic classifications is listed. (DOCX)
