# Supplementary material for: Diagnostic Performance of Magnifying Endoscopy for Helicobacter pylori Infection: A Meta-Analysis
Source: PLoS One. 2016 Dec 19;11(12):e0168201. doi: 10.1371/journal.pone.0168201 (PMC5167261; doi:10.1371/journal.pone.0168201)
Supplement: S1 Text — (DOCX) [file pone.0168201.s002.docx]

**S1 Text**

**The descriptions of diagnostic classifications used in the included studies.**

1. wDRP, iDRP: wDRP, well-defined ridge pattern; iDRP, ill-defined ridge pattern.
2. Z0-3: Z0, collecting venules, with true capillaries forming a network, and gastric pits resembling pinholes; Z1,irregular true capillaries but no collecting venules observed; Z2, white gastric pits and sulci, with neither collecting venules nor true capillaries being seen;Z3, dilated pits with surrounding redness.
3. R.I.O: R (regular), regularity in venulae size, visible second or third order branches, and a uniform distance between venulae; I (irregular), irregularity in size, inability to observe second or third order branches, and a lack of a uniform distance between the collecting venulae, with venulae sometimes fused to adjacent venulae and sometimes lying horizontally; O (obscured), no collecting venulae were visible.
4. R.I.D: R (regular), with diameter of minor venules being 0.4-0.5 mm and regular spider-like and jellyfish-like arrangement; I (irregular), decrease in quantity of collecting venules could be unclearly found with irregular arrangement; D (disappeared), collecting venules could not be found under magnifying endoscope.
5. Fundic type and nonfundic type: Fundic type, a flat pinpoint feature; nonfundic type, an irregular granular or papillary pattern.
6. Type1-3: Type 1, honeycomb−type subepithelial capillary network (SECN) with regular arrangement of collecting venules and regular, round pits; type 2, honeycomb−type SECN with regular, round pits, but loss of collecting venules; type 3, loss of normal SECN and collecting venules, with enlarged white pits surrounded by erythema.
7. CZ0-2: CZ0, regularly arranged pinpoint gastric pit openings; CZ1, gastric pit openings with comma or rod-like shape and without a uniform distance between pits; CZ2, distorted mucosal architecture with dilated sulci and inapparent pit openings.
8. CZA0-2: CZA0, regularly arranged ridge pattern; CZA1, irregular ridges with dilated sulci, general mucosal architecture protected; CZA2, disrupted mucosal pattern with villi-like projections.
9. Normal+Type1-3: Normal pattern: small, round pits surrounded by SECNs, which are regularly interspersed with collecting venules; Type 1: slightly enlarged, round pit with unclear or irregular SECNs; Type 2: obviously enlarged, oval or prolonged pit with increased density of irregular vessels; Type 3: well-demarcated, oval or tubulovillous pit with clearly visible coiled or wavy vessels.
10. COs: COs is short for “the whiteness of crypt openings”, which is classified as white-edged dark spot COs (a round dark spot bordered by white), white COs (pure white COs without a dark spot) and dense white pit COs (densely white COs resembling snowballs).
11. Type A-E: Type A, continuous short rod-like pits or closed ring shape with regular microvasculature inside pits; Type B, elongated and tortuous open branch-like pits with regular microvasculature; Type C, dilated pits with a thin or unclear epithelial layer and increased branching microvasculature; Type D, villus-like appearance or blue crest sign; Type E, circumscribed lesions characterized by the disappearance of normal pits and the appearance of new tumor vessels.
